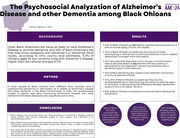# The Psychosocial Analyzation of Alzheimer’s Disease and other Dementia among Black Ohioans

**DOI:** 10.1002/alz.090376

**Published:** 2025-01-09

**Authors:** Camren J Harris

**Affiliations:** ^1^ Alzheimer’s Association, Columbus, OH USA

## Abstract

**Background:**

Older Black Americans are twice as likely to have Alzheimer’s Disease or another dementia, and 65% of Black Americans say that they know somebody with Alzheimer’s or dementia. More locally, according to Ohio county level estimates, 11.3% of Ohioans aged 65 are currently living with Alzheimer’s Disease. Higher than the national average 10.7%

**Method:**

A small sample size of Black Ohioans from different counties took a questionnaire pertaining to information as it relates to Alzheimer’s Disease and other dementia in the Black Communities in Ohio.

**Result:**

82% of Black Ohioans surveyed know someone who has been diagnosed with Alzheimer’s Disease or related dementia. 64% of Black Ohioans do ***not*** believe cognitive decline issues are a part of normal aging. Only 35% of Black Ohioans surveyed say they are very knowledgeable about dementia. 87% of Black Ohioans surveyed are concerned about developing Alzheimer’s. 94% of Black Ohioans surveyed believe that the lack of access to resources is a reason why Black Americans are more likely to be diagnosed with Alzheimer’s Disease. 62% of Black Ohioans surveyed say they would consider participating in clinical trial concerning Alzheimer’s and/or related dementias if provided more information. 94% recorded that they have not (in the last 6 months) become aware of ***any*** elected officials creating or advocating for any legislation concerning cognitive care or dementia support and treatment. 52% believe dementia care, support and treatment should be a priority for elected officials.

**Conclusion:**

Black Ohioans are concerned about the rising prevalence of Alzheimer’s Disease, however social barriers that prevent equitable access to care, support and treatment is of overwhelming concern. Additionally, Black Ohioans are interested in participating in clinical trials but due to the lack of resourceful information presented and/or provided concerning clinical trials to Black communities, further creates reluctance to participate. Lastly, more than half of the Black Ohioans surveyed are simply unaware of the public policy issues and priorities concerning cognitive decline, but would like for elected officials (federal, state and local) to be more intentional about utilizing public policy to build a better dementia infrastructure for those affected by Alzheimer’s Disease and other dementias.